# Antibacterial activity identification of pCM19 and pCM12 derived from hGlyrichin

**DOI:** 10.1186/s40064-016-3025-4

**Published:** 2016-08-22

**Authors:** Jibin Sha, Chenggang Zhang

**Affiliations:** 1 Beijing Institute of Radiation Medicine, 27 Taiping Road, Haidian District, Beijing, 10085 China; 2 School of Basic Sports Science, Shandong Sports University, Jinan, China

**Keywords:** Antimicrobial peptides, hGlyrichin, Hemolytic effect

## Abstract

**Background:**

hGlyrichin is a novel human antimicrobial peptide rich in glycine. The previous study of known human antimicrobial peptides indicated that in an eligible range, the greater corresponding antibacterial activity was consisted with the shorter peptide sequence.

**Findings:**

Two peptides named pCM19 and pCM12 were synthesized and the antibacterial activity assay results showed that these peptides exhibited strong antibacterial activity that was inversely proportional to the length of the peptide. Despite the effective inhibition of bacterial growth, the synthetic peptides showed no hemolytic effect on human red blood cells.

**Conclusions:**

Taken together, these two peptides derived from hGlyrichin both have strong antibacterial activity and are not toxic to human somatic cells.

## Background

Mature antimicrobial peptides are usually processed from their precursors (Vassilevski et al. 2008; Brown and Hancock 2006). The post-translational modifications of peptides include proteolysis, glycosylation, C-terminal amidation, amino acid isomerization, and halogenation. After the mature peptide is formed by a cleavage process, it is antimicrobially active.

By comparing the sequences of the defensin family from plants, insects, birds, mammals, and other species, Yount and Yeaman identified a highly conserved three-dimensional structure containing disulfide bonds, known as the γ core motif (Yeaman and Yount 2003). This special bi-directional amino acid sequence motif contains two anti-parallel β-sheet structures and a short coil insert sequence, and exists in all species, suggesting that the defensins from different species may be derived from a common precursor (Ganz 2003; Selsted and Ouellette 2005). The results suggest that the γ core motif may be the functional domain of the defensin family.

The cathelicidin family is known to contain 30 members. According to its structural characteristics, the cathelicidin family can be classified into four subfamilies (Lehrer and Ganz 2002; Bals and Wilson 2003). The cathelin-like structure in the C segment is a common feature in all members of this family. At present, the most active sequence in the cathelicidin family is LL-37, which is the shortest sequence and is expressed by human leukocytes.

Furthermore, analysis of the antimicrobial activities of peptides from bactenecin showed that their antibacterial activity was increased and their antibacterial spectrum was broadened when the peptide length was shortened from 12 amino acids to eight amino acids (Hilpert et al. 2005).

hGlyrichin is a new peptide isolated from a human fetal liver cDNA library. The amino acid sequence of hGlyrichin is rich in glycine (Sha et al. 2012). Chung et al. named this same gene Romo1 (reactive oxygen species modulator 1) in 2006 and showed that it encodes a protein that is identical to hGlyrichin (Chung et al. 2006). Bioinformatics analysis indicated it has 79 amino acid residues, >21 % of which are glycine, and the pI value is 9.58. Secondary structure analysis shows that hGlyrichin is an amphiphilic molecule containing mainly β-sheets with alternating hydrophilic and hydrophobic regions. This characteristic is highly consistent with the structural characteristics of cationic antimicrobial peptides. Functional analysis of the synthetic peptides revealed that hGlyrichin is an important member of a potentially novel human cationic antimicrobial peptide family, which may act on the bacterial membrane (Sha et al. 2012).

In order to verify whether or not hGlyrichin was in accord with the regular pattern mentioned above: in an eligible range, the shorter the peptide sequence is, the greater the corresponding antibacterial activity is (Kindrachuk 2010; Hwang and Vogel 1998; Sørensen et al. 2001), we compared the antibacterial activity and hemolytic side effects of two peptides derived from hGlyrichin.

## Methods

### Bacteria strains and cells

*Escherichia coli* (*E. coli*) BL21 and ampicillin-resistant (*Amp*^*R*^) *E. coli* BL21 (*E. coli* BL21 transformed with the pET-22b+ plasmid which carried the ampicillin-resistant gene) were maintained in our laboratory. *Staphylococcus aureus* (*S. aureus*, ATCC 25923), ampicillin-resistant *S. aureus* (*S. aureus Amp*^*R*^, ATCC BAA-44), and *Salmonella* Typhi (*S.* Typhi, ATCC 19430) were maintained at the Institute of Microbiology of Military Academy of Medical Sciences. The antimicrobial activity assays were completed in a specialized laboratory. Red blood cells were collected from healthy adults at the 307 hospital laboratory. All participants gave informed consent to participate in the study.

### Peptide synthesis

All peptides were synthesized by GL Biochem (Shanghai, China), and their sequences are as follows: pCM19 (contains 19 amino acids from positions 42 to 60, which had been authenticated as crucial fragment for the antibacterial activity of hGlyrichin in our previous study) (Sha et al. 2012), CLRIGMRGRELMGGIGKTM; pCM12 (12 amino acids of pCM19 from which 7 amino acids at the carboxyl terminal were removed), CLRIGMRGRELM; Flexible fragment (FF), a GIG array that is more like a flexible unit and represents the portion that is different between pCM19 and pCM12, GGIGKTM; Positive control (PC, a peptide segment based on the P2 polypeptide of the human neutrophil bactericidal permeability increasing protein, BPI) (Barker et al. 2000), SKISGKWKAQKRFLKMSGNFGC; Random control (RC, a random array of pCM12), GICRLMMRRGLE; pCM11 (pCM12 with the last amino acid residue removed), CLRIGMRGREL. All of the peptides were diluted to 20 mg/ml with sterile deionized water as stock solutions and stored at −20 °C.

### Experimental methods

#### Bacteria colony counting method

A total of 5 µl of the bacteria solution at *OD*_600_ = 0.3 containing *E. coli* BL21, *E. coli* BL21 *Amp*^*R*^, *S. aureus*, *S. aureus Amp*^*R*^, or *S.* Typhi, were diluted to 20 µl with LB medium. A total of 10 µl of the peptide solution containing 100 µg of the peptides (pCM19, pCM12, pCM11, flexible peptide, randomized control peptide, or the positive control peptide), 10 µl of Amp solution (containing Amp 100 µg), or 10 µl of double-distilled water was added, mixed well, and incubated at 37 °C for 2 h. Twenty microliters of the culture was diluted in LB (final volume 1 ml) and was evenly coated on an LB plate, inverted and incubated at 37 °C for 12–18 h and then the bacterial colonies were counted.

#### Bacterial growth curve method

Eight microliters of bacterial solution at *OD*_600_ = 2.5 containing *E. coli* BL21, *E. coli* BL21 *Amp*^*R*^, *S. aureus*, *S. aureus Amp*^*R*^, or *S.* Typhi were mixed with 12 µl of the peptide solution containing 120 µg of the peptide (pCM19, pCM12, pCM11, flexible peptide, randomized control peptide, or the positive control peptide), or 12 µl Amp solution (containing 120 µg Amp), or 12 µl of double-distilled water. The mixtures were mixed well, and incubated at 37 °C for 1 h. LB was added to bring the volume up to 4 ml, and the incubation was continued at 37 °C and 175 rpm for 10 h. Fifty microliters of each samples were taken every 2 h, and the absorbance of each sample at 600 nm was determined by spectrophotometry. The bacterial growth curve was calculated.

### In vitro hemolysis assay

Blood samples from healthy donors were treated with heparin to minimize clotting. The blood cell was washed three times with normal saline, and 8 µl samples were diluted to 100 µl in normal saline. The diluted blood cell (100 µl) was incubated with 100 µl of pCM19 (3000 µg/ml) for 1 h at 37 °C; then the same volume diluted blood cell was incubated with 100 µl of pCM12 (100, 200, 500, 1000, 2000, 3000 µg/ml) for 1 h at 37 °C. Centrifugation was carried out at 1000 rpm for 5 min at 4 °C. Each treatment was performed in triplicate. The absorbance of each sample was measured at 570 nm using a microplate reader. The control group contained samples treated with 100 µl normal saline (negative control), 0.1 % Triton X-100 (positive control), or ampicillin (3000 µg/ml, traditional antibiotic) respectively, and the controls were treated in triplicate.

## Results

### The antibacterial activity identification and comparison of the peptide pCM19 and pCM12

#### Analysis of antibacterial activity based on bacteria colony counting

**Fig. 1:**
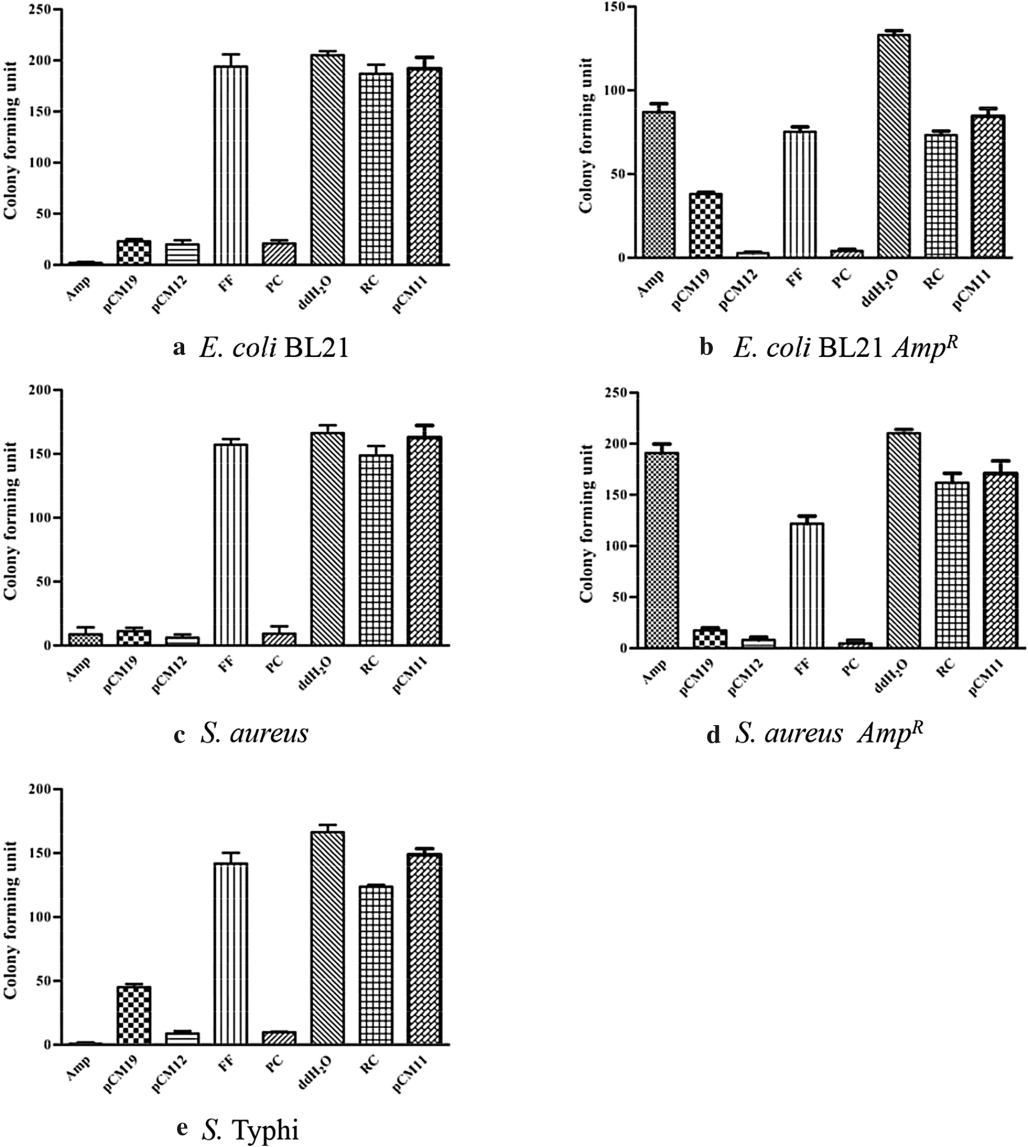
Analysis of the antibacterial activity of different peptides by bacteria colony counting. Antibacterial effect of different peptide segments was compared on *E. coli* BL21 (**a**), *E. coli* BL21 *Amp* ^*R*^ (**b**), *S. aureus* (**c**), *S. aureus Amp* ^*R*^ (**d**) and *S.* Typhi (**e**). Data are expressed as the mean ± SD (n = 5)

As shown in Fig. 1, the pCM19 and pCM12 peptides effectively inhibited the growth of both Gram-negative bacterium (*E. coli* BL21 and *S.* Typhi) and Gram-positive bacterium (*S. aureus*). Most importantly, both of these peptides showed good inhibition and killing of the ampicillin-resistant bacteria *E. coli* BL21 *Amp*^*R*^ and *S. aureus Amp*^*R*^. A comparison of the overall antimicrobial activities showed that pCM12 had stronger antimicrobial activity than pCM19. Additionally, the antimicrobial activity of pCM12 was similar or slightly better than the positive control peptide, while the flexible peptide from the C-terminal GIG region of pCM19, almost completely lost the antibacterial activity. In addition, when the amino acid sequence of pCM12 was altered by random rearrangement, the RC peptide almost completely lost its bactericidal activity, which suggested that a particular amino acid sequence (which called primary structure of the peptide) was desicive for its antibacterial activity. However, when the last amino acid of pCM12 was removed, the pCM11 peptide also lost its antibacterial activity, indicated that the Met amino acid residue was significant for maintaining the nature activity of the peptide.

#### Analysis of the antibacterial activity based on the bacterial growth curve

**Fig. 2:**
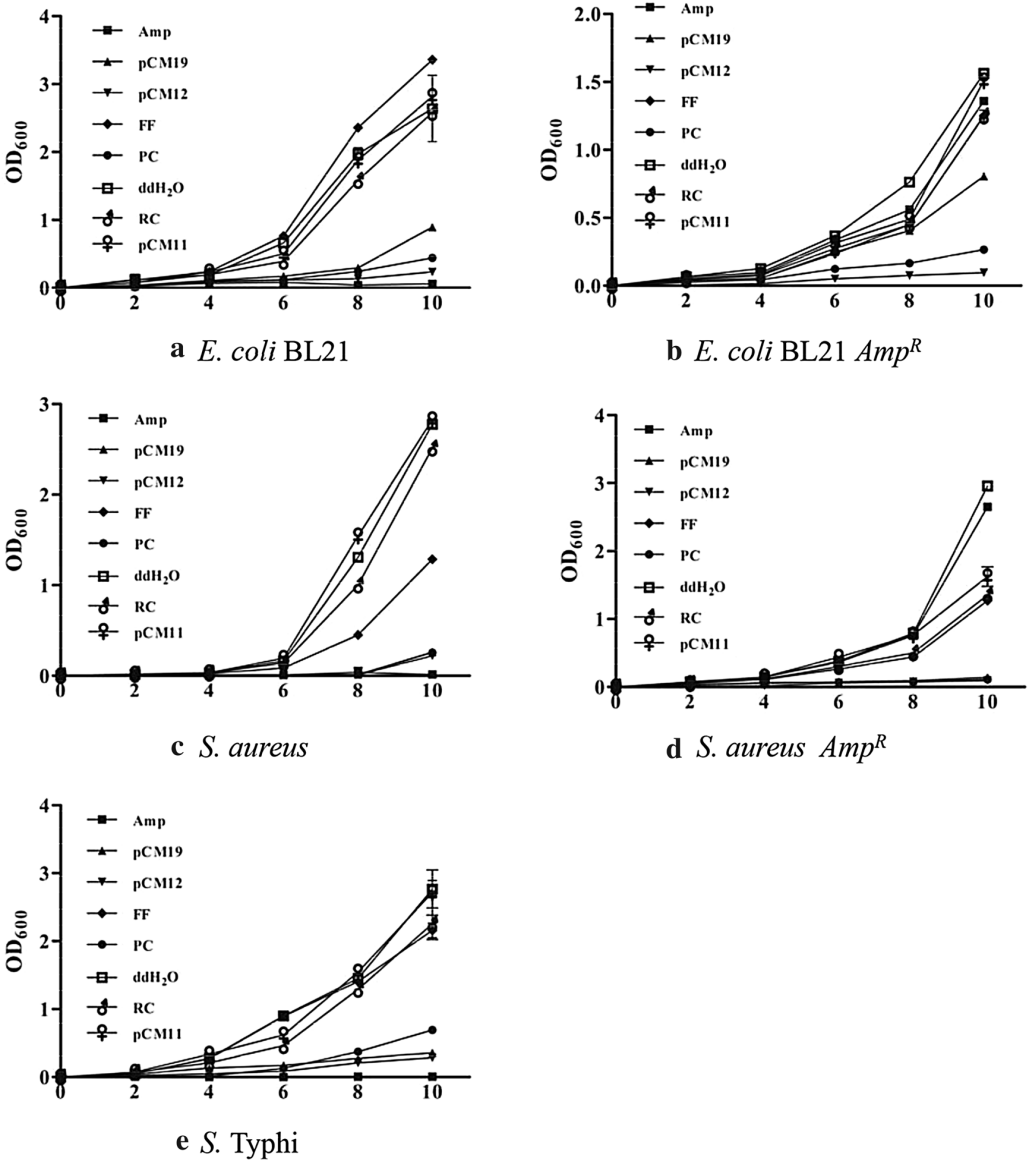
Analysis of the antibacterial activity of different peptides by bacterial growth curve. Antibacterial effect of different peptide segments was compared on *E. coli* BL21 (**a**), *E. coli* BL21 *Amp* ^*R*^ (**b**), *S. aureus* (**c**), *S. aureus Amp* ^*R*^ (**d**) and *S.* Typhi (**e**). Data are expressed as the mean ± SD (n = 5)

As shown in Fig. 2, during the first 10 h incubation, the antibacterial effects of pCM19 and pCM12 remained stable. The data obtained from the growth inhibition and killing analysis on *E. coli* BL21, *S. aureus*, and *S.* Typhi as well as the ampicillin-resistant bacteria *E. coli* BL21 *AmpR* and *S. aureus AmpR* were essentially consistent with the results of colony counting. A comparison of the overall antimicrobial activities showed that pCM12 had more potent antimicrobial activity than pCM19. Compared with the positive control peptide, the antimicrobial activity of pCM12 was similar or slightly better, while neither the flexible peptide from the C-terminal GIG region of pCM19, nor pCM11 and the random control peptide showed no obvious antimicrobial activities.

Compared with pCM19, the peptide sequence of pCM12 is shorter, which will significantly reduce the difficulty and the cost associated with its synthesis, and make this peptide more amenable to practical applications.

### Results of the in vitro hemolysis assay of the peptide pCM19 and pCM12

Following the incubation of human blood cells with pCM19 or pCM12, the *OD*_*570*_ value was measured. pCM12 and pCM19, even at a much higher concentration than the effective antibacterial dose, caused no damage to the red cell membranes and were comparable to the negative control groups that were treated with water or ampicillin (Table 1). Thus, these two peptides derived from hGlyrichin exhibited good selectivity between the bacterial membranes and normal human cell membranes.

**Table 1:** In vitro hemolysis assay of the peptides pCM19 and pCM12

Solution	Sample 1	Sample 2	Sample 3	Mean	SD
Triton X-100	4.949	6.000	5.552	5.500	0.5274
pCM19 (100 µg/ml)	0.088	0.131	0.093	0.104	0.0235
pCM19 (3000 µg/ml)	0.131	0.127	0.142	0.133	0.0078
pCM12 (100 µg/ml)	0.105	0.123	0.104	0.111	0.0107
pCM12 (200 µg/ml)	0.114	0.116	0.122	0.117	0.0042
pCM12 (500 µg/ml)	0.120	0.134	0.136	0.130	0.0087
pCM12 (1000 µg/ml)	0.140	0.141	0.141	0.141	0.0006
pCM12 (2000 µg/ml)	0.140	0.141	0.140	0.140	0.0006
pCM12 (3000 µg/ml)	0.105	0.123	0.104	0.111	0.0107
Normal saline	0.187	0.162	0.176	0.175	0.0125
Amp (3000 µg/ml)	0.076	0.152	0.103	0.110	0.0535

## Discussion

Antimicrobial peptides are highly conserved molecules despite of the diversities among their sequences and structures. Almost more than 2000 antimicrobial peptides have been identified, and they can be classified into a few conformational models (Powers and Hancock 2003; Hilpert et al. 2006). Newly identified antimicrobial peptides, especially human origined cationic antimicrobial peptides, have become an important platform for the discovery of novel antimicrobial agents for emerging drug-resistant bacteria.

hGlyrichin is a human cationic antimicrobial peptide encoded by an evolutionarily highly conserved gene. In our previous study, we confirmed the 19 amino acid peptide (pCM19) at positions 42–60 of hGlyrichin is crucial for its antibacterial activity. Thus, we wanted to investigate whether it consist with the same regular pattern summarized before: in an eligible range, the shorter the peptide sequence length, the greater the corresponding antibacterial activity. So we synthesized the peptide pCM12, and compared its antibacterial activity with pCM19. pCM19 and pCM12 were derived from hGlyrichin and showed significant inhibition and killing activities against the laboratory-engineered bacteria, Gram-positive bacteria, Gram-negative bacteria, and ampicillin-resistant bacteria. The results indicated that pCM12 showed a slightly better antimicrobial activity than that of pCM19 for most target bacteria. The inactivity of the random control peptide and pCM11 confirmed the importance of the correct amino acid sequence and the last amino acid for the observed antibacterial activity. The in vitro hemolysis assay showed that the peptides from hGlyrichin had a high degree of selectivity for bacterial membrane. Even at higher concentrations, they did not damage the normal red blood cells.

The additional results indicated that after the N-terminal cysteine of pCM19 is removed, its antibacterial activity has almost completely lost, which suggests that cysteine also played a crucial role in maintaining the bactericidal activity of this peptide. It was known that the number of net positive charges of the antimicrobial peptide was an important structure element which affected the interaction between cationic antimicrobial peptides and the negatively charged phospholipid membranes. However, based on the known structure pCM19, the synthetic peptides with increased net positive charge number did not show distinct improvement of antibacterial activity (unpublished data, Sha et al.). These results suggest that, in killing bacteria, the integrity of the bactericidal domain plays more important role than the overall charge of the peptide. Due to the results above, the peptides of hGlyrichin have the potential to be developed into a new type of safe and effective antibacterial agent.

In fact, the structure of the antimicrobial peptide and its activity are governed by its degree of cationization (positive charge content), amphiphilic characteristics, hydrophobic characteristics, structural tendency, amino acid sequence composition, angular degree, and amphiphilic balance, or cation/hydrophobic balance, that is the optimum ratio between the number of cations and the peptide’s hydrophobicity (Yeaman and Yount 2003; Barker et al. 2000; Zasloff et al. 1988; Takahashi et al. 2010; Bechinger 2011; Palermo and Kuroda 2010). To further clarify the relationships between these primary structure parameters and the antimicrobial activities of the peptides, high-throughput methods such as combinatorial chemistry (Hilpert et al. 2006; Fjell et al. 2012) should be helpful.

## Conclusions

In this study, based on the in-depth antibacterial activity analysis of the peptides derived from hGlyrichin, we concluded that, as compared with the 19 amino acid peptide pCM19, the shorter peptide pCM12 is more effective in killing bacteria, in particular, the bacteria with antibiotic resistance and high virulence. This conclusion is also consistent with what has been observed with defensins, cathlicidins, and bactenecins. Finally, the inactivity of pCM11 demonstrates the critical role of the last Met residue of pCM12 in killing bacteria.
